# Population-based resequencing revealed an ancestral winter group of cultivated flax: implication for flax domestication processes

**DOI:** 10.1002/ece3.101

**Published:** 2012-03

**Authors:** Yong-Bi Fu

**Affiliations:** Plant Gene Resources of Canada, Saskatoon Research Centre, Agriculture and Agri-Food Canada,107 Science Place, Saskatoon, SK S7N 0X2, Canada

**Keywords:** Crop domestication, cultivated flax, pale flax, sequence variation, winter hardiness

## Abstract

Cultivated flax (*Linum usitatissimum* L.) is the earliest oil and fiber crop and its early domestication history may involve multiple events of domestication for oil, fiber, capsular indehiscence, and winter hardiness. Genetic studies have demonstrated that winter cultivated flax is closely related to oil and fiber cultivated flax and shows little relatedness to its progenitor, pale flax (*L. bienne* Mill.), but winter hardiness is one major characteristic of pale flax. Here, we assessed the genetic relationships of 48 *Linum* samples representing pale flax and four trait-specific groups of cultivated flax (dehiscent, fiber, oil, and winter) through population-based resequencing at 24 genomic regions, and revealed a winter group of cultivated flax that displayed close relatedness to the pale flax samples. Overall, the cultivated flax showed a 27% reduction of nucleotide diversity when compared with the pale flax. Recombination frequently occurred at these sampled genomic regions, but the signal of selection and bottleneck was relatively weak. These findings provide some insight into the impact and processes of flax domestication and are significant for expanding our knowledge about early flax domestication, particularly for winter hardiness.

## Introduction

Cultivated flax (*Linum usitatissimum* L.) is a multiple purpose crop being utilized for oil and fiber and its early domestication history may involve multiple events of domestication for oil, fiber, capsular indehiscence, and winter hardiness (Allaby et al. 2005; Fu et al. 2012). Recent discovery of the early domestication event for capsular indehiscence (Fu 2011) not only supports this argument, but also stimulates more searches for clues on the early domestication history. Dehiscent flax (i.e., cultivated flax with spontaneously opening capsules) is genetically unique and displays close relatedness to its wild progenitor, pale flax (*L. bienne* Mill. or previously *L. usitatissimum* L. subsp. *angustifolium* [Huds.] hell.; Hammer 1986). In contrast, winter flax (i.e., cultivated flax with a vernalization requirement) is closely related to oil or fiber forms of cultivated flax and distantly related to its progenitor (Fu 2011). Possibly, these findings are clouded with inadequate sampling of diverse flax and/or limited genomic sampling with insufficient molecular markers (Uysal et al. 2010; Fu et al. 2012). Given the facts that capsular dehiscence and winter hardiness are two major characteristics of pale flax and that cultivated flax was spread from the warm Near East to the cold Europe (Maier and Schlichtherle 2011), we hypothesized that winter hardiness was among those flax traits that human domesticated early (Fu 2011). We further reasoned that sampling more ancestral genetic diversity (Charlesworth 2010) may help to reveal closer relatedness between winter flax and pale flax, as some winter flax may have experienced differential domestication pressure over time and still carry more ancestral polymorphism.

Assessments of genetic relationships among various groups of cultivated flax with unique domestication-associated traits can provide insights into its domestication paths, as trait-specific groups should carry unique genetic traces of plant domestication accumulated over time (Fu 2011) and groups with early versus recent domesticated traits may display different levels of genetic relatedness to its progenitor (Zohary 1999). Early efforts were made to group cultivated flax based on specific flax traits (Elladi 1940; Dillman 1953; Kulpa and Danert 1962) to facilitate flax germplasm conservation, utilization, and research. The commonly referred or applied groups of cultivated flax are oil flax (i.e., cultivated flax with improved oil composition), fiber flax (i.e., cultivated flax with improved fiber characters), dehiscent flax, and winter flax (Diederichsen and Fu 2006). Interestingly, these four traits are associated with flax domestication (Hammer 1984; Uysal et al. 2012). Generally, cultivated flax is an annual, self-pollinating crop, has variable seed dormancy, grows fast with large variation in the generative plant parts, and has early flowering, almost indehiscent capsules, and large seeds. However, pale flax is a winter annual or perennial plant with narrow leaves and dehiscent capsules, and usually displays large variation in the vegetative plant parts and variable growth habit (Diederichsen and Hammer 1995; Uysal et al. 2012).

Pale flax has been identified as the wild progenitor of cultivated flax (Tammes 1928; Gill 1987; Fu et al. 2002; Fu and Allaby 2010). The archeological records of pale flax were obtained first from Tell Abu Hureyra in northern Syria (11,200–10,500 years ago) (Hillman 1975) and then throughout the Near East by the 8th millennium BC (Zohary and Hopf 2000). The archeological finds from Tell Ramad in Syria (9000 years ago) revealed the first occurrence of cultivated forms of flax with an increase in seed size (van Zeist and Bakker–Heeres 1975). archeological evidence also existed for flax spreading from the Near East to Europe and the Nile Valley (Maier and Schlichtherle 2011). The recent archeological finds in southwest Germany revealed larger flax seeds in the earlier, than later, phase of the Late Neolithic (4000–2500 cal. BC) (Herbig and Maier 2011). The flax varieties that spread into the Danube valley were winter oil varieties. However, summer fiber varieties developed in eastern Europe also spread into central Europe and replaced the original varieties (Helbaek 1959; Diederichsen and Hammer 1995). All modern fiber varieties may have originated from eastern Europe (Helbaek 1959). Nowadays, flax is cultivated in more than 60 countries around the world (Fu 2005). The rest of the early history of flax domestication, however, remains unknown (Zohary and Hopf 2000; Allaby et al. 2005).

The objective of this study was to assess genetic diversity and genetic relationships of 48 *Linum* samples representing pale flax and four trait-specific groups of cultivated flax (dehiscent, fiber, oil, and winter flax) through population-based resequencing at 24 genomic regions. Recent development of genomic resources in *Linum* species through Roche 454 pyrosequencing technology (454 Life Sciences, Branford, CT) (Fu and Peterson 2012) made the genetic sampling of flax genome more feasible than before.

## Materials and Methods

All flax accessions studied here were obtained from the flax collection at the Plant Genetic Resources of Canada (PGRC; Table 1). They include 10 pale flax accessions from Turkey and Greece and 38 cultivated flax accessions from 26 countries. The selection of pale flax accessions is limited due to the lack of widely distributed pale flax germplasm and the selected ones represent only the central part of its natural distribution spanning the western Europe and the Mediterranean, north Africa, western and southern Asia, and the Caucasus regions (Diederichsen and Hammer 1995). The cultivated flax accessions were selected based on previous phenotypic and genetic studies (e.g., see Diederichsen and Fu 2006) to represent four major intraspecific groups of cultivated flax (dehiscent, fiber, oil, and winter flax). The winter flax accessions sampled cultivated flax developed with winter hardiness from eight countries. The dehiscent flax accessions represent the primitive form of cultivated flax with dehiscent capsules and have been long accumulated from flax cultivation in the cultivated flax gene pool (Hegi 1925). For this study, the dehiscent flax accessions were empirically verified for capsular dehiscence, and the selected pale flax accessions were assessed for their taxonomic identity in the greenhouse.

**Table 1:** List of 48 accessions of wild and cultivated flax sequenced, with their species/group, origin country, *sad2* haplotype, inferred cluster, and label.

CN^1^	Species/group^2^	Description^3^	Origin^3^	H- *sad2*^3^	Cluster^3^	Label^4^
T19719	Lb	Island of Evia	GRC	I	C2	P1
T19716	Lb	Rhodes airport	GRC	I	C2	P2
113606	Lb	Samsun	TUR	XI	C3	P3
113622	Lb	Antalya	TUR	II	C1	P4
113627	Lb	Sinop	TUR	IV	C2	P5
113628	Lb	Karabük	TUR	V	C2	P6
113630	Lb	Kastamonu	TUR	IX	C3	P7
113633	Lb	Zonguldak	TUR	IX	C3	P8
113638	Lb	Çanakkale	TUR	II	C1	P9
113642	Lb	Trabzon	TUR	XI	C3	P10
97606	Lu-d		ESP	III	C1	D1
100852	Lu-d	Grandal	PRT	III	C1	D2
100910	Lu-d	Grandal	PRT	III	C1	D3
97769	Lu-d	Abertico	PRT	III	C1	D4
97473	Lu-d		RUS	III	C1	D5
98833	Lu-d		RUS	III	C1	D6
97605	Lu-d		RUS	III	C1	D7
100837	Lu-d		TUR	III	C1	D8
98986	Lu-f	Crista	BEL	IX	C4	F1
101017	Lu-f	Baladi	CHN	VIII	C4	F2
101388	Lu-f	Saskai	CZE	IX	C4	F3
98475	Lu-f	Flachskopf	DEU	VIII	C4	F4
101111	Lu-f	Viking	FRA	X	C4	F5
98946	Lu-f	Talmune Fiber	NLD	IX	C4	F6
101120	Lu-f	Liana	POL	X	C4	F7
97325	Lu-f	Kotowiecki	POL	VIII	C4	F8
18991	Lu-f	Nike	RUS	IX	C4	F9
101397	Lu-f	Pskovski 2976	UKR	X	C4	F10
18974	Lu-o	CDC Bethune	CAN	X	C4	O1
100832	Lu-o	Barbarigo	CZE	VII	C4	O2
101171	Lu-o	Hermes	FRA	X	C4	O3
18989	Lu-o	Atalante	FRA	IX	C4	O4
101265	Lu-o	Amason	GBR	VI	C4	O5
98256	Lu-o	Arreveti	IND	VI	C4	O6
97888	Lu-o	Tomagoan	IRN	IX	C3	O7
101268	Lu-o	Raisa	NLD	IX	C4	O8
100917	Lu-o	Raluga	ROM	IX	C4	O9
33399	Lu-o	Bison	USA	X	C4	O10
98178	Lu-w	1285-S	AFG	X	C3	W1
96915	Lu-w	Uruguay 36/49	AUS	VI	C4	W2
97009	Lu-w	Beladi Y 6903	EGY	IX	C3	W3
97004	Lu-w		ETH	VI	C4	W4
98509	Lu-w		ISR	IX	C4	W5
97102	Lu-w		PAK	IX	C4	W6
96960	Lu-w		SYR	IX	C3	W7
96848	Lu-w		TUR	IX	C4	W8
100828	Lu-w		TUR	X	C3	W9
100829	Lu-w		TUR	VIII	C4	W10

1 CN, Canadian National accession number at Plant Gene Resources of Canada (PGRC), Saskatoon, Canada; T, temporary number for accessions that were acquired, but not yet added to the PGRC germplasm collection.

2 Lb, *Linum bienne*; Lu, *Linum usitatissimum*. Four letters (d, f, o, w) represent four trait-specific groups of cultivated flax (dehiscent, fiber, oil, winter), respectively.

3 Description of an accession includes the record, if available, for varietal or local name, location, and feature; Origin, origin of country; H- *sad2*, *sad2* haplotype obtained from Fu et al. (2012); four clusters inferred using the BEAST program.

4 Accession label is consisted of the first letter for species (P, *L. bienne)* or group of cultivated flax (D, dehiscence; F, fiber; O, oil; W, winter), followed by the numbers distinguishing among accessions within a species or group.

### DNA extraction

Plants were grown from seed for two to three weeks for cultivated flax and up to two months for pale flax in a greenhouse at the Saskatoon Research Centre, Agriculture and Agri-Food Canada. Young leaves were individually collected, freeze-dried, and stored at –20°C. A freeze-dried leaf sample of one individual plant from each accession was selected, and its genomic DNA was extracted with the DNEasy Plant Mini kit (Qiagen, Mississauga, Ontario, Canada). Extracted DNA was quantified with a Thermo Scientific NanoDrop 8000 spectrometer (Fisher Scientific Canada, Toronto, Ontario, Canada).

### PCR and Sanger resequencing

Sanger resequencing was performed on 24 confirmed contigs available in the *Linum* genomic resources developed through the Roche 454 pyrosequencing technology (Fu and Peterson 2012). The contig selection was mainly based on its polymorphism and quality, as gene annotations on all developed contigs were incomplete and unverified (Fu and Peterson 2012). The PCR primers for 24 loci were designed using the on-line Primer Quest tool (Integrated DNA Technologies, Coralville, IA) (Table 2). The conditions for PCR were: 1× KAPA 2G Buffer A containing 1.5 mM MgCl_2_ (KAPA Biosystems, Woburn, MA), 1× KAPA Enhancer 1, 0.2 mM each dNTP, 0.4 pmol/µl each forward and reverse primers, 100 ng of the same genomic DNA template samples as used above for next-generation sequencing, and 0.5 U KAPA 2G Robust polymerase in a final volume of 25 µl; touchdown PCR cycled at 95°C for 3 min followed by 10 cycles of 95°C for 10 sec, 60°C decreasing 0.5°C per cycle for 15 sec, 72°C for 30 sec followed by 25 cycles of 95°C for 10 sec, 55°C for 15 sec, 72°C for 20 sec, followed by a final extension of 72°C for 30 sec. A 3-µl sample of each PCR product was separated on 1.5% agarose for 2 h at 120 V. PCR was performed on either a DYAD or PTC-200 thermocycler (Bio Rad, Mississauga, Ontario, Canada). PCR products were cleaned following the method outlined by Rosenthal et al. (1993) and submitted for Sanger sequencing at the DNA Technologies Laboratory at the Canadian National Research Council's Plant Biotechnology Institute (Saskatoon, Saskatchewan, Canada).

**Table 2:** List of 24 primer pairs used by Sanger resequencing of 24 contigs representing polymorphic genomic regions in the 48 *Linum* accessions, along with the polymorphism and gene annotation information.

Primer^1^	Sequence (5′>3′)^1^	Tm (°C)^2^	CL^2^	Ts^2^	Nh^2^	π^2^	Scaffold|GO^2^
031B/A	CTCATCTTCTTCTTCCTTACATCTGACG/AACAGGACGCCCGAATGAATTG	56.6/58.1	346	10	5	0.0064	sc453|gn
049B/A	TGCAGGTGTGCCTGAATCTGACAT/AACAGGCCTTGGTGGGTCTAATGA	60.8/60.3	334	13	8	0.0078	sc401|gn
071B/A	AGGACCATTGTGTTGCAAGCATCC/CCAATCATCTTTGGATCTGTCCAGG	60.2/57.5	283	15	17	0.0079	sc297|g13204
145B/A	GGACAAGGGTTCATTTCGTGAAAGCG/AGTRGCATCCTCGGAACTTCTCTT	60.4/58.6	364	3	5	0.0033	sc530|g23272
151B/A	ACAAAGACACCAATGCTCCCTCCT/TCCRGGCATGGAAAGATATTAAGT	60.5/55.0	363	10	5	0.0077	sc142|gn
204B/A	TGTTTATTGACATAATTGGACGAAA/AACGCCCTTACGAATGRACAYTA	51.4/56.5	225	4	5	0.0042	sc181|gn
221B/A	TGTAGGGATAGCGAACGATAGTAAC/CCCTTTCATTCCACGGTAGCAA	55.6/57.5	370	12	8	0.0114	sc186|gn
242B/A	ACTCTAACAGACAAGGCCACCGAT/GCCATACAAGCATGGATCCTGTCA	59.9/59.2	304	4	9	0.0061	sc475|g20873
246B/A	AATTCAGGGAGCGACACAGCCAGA/CAACCGTCGACAAGTTGGCAAGAA	62.5/60.0	259	5	5	0.0029	sc1078|g37692
281B/A	AACTCTGCTCTCATTCCTGCCGAA/ACCTCGAGTACATCTCGTTCGCAT	60.3/59.7	285	7	6	0.0060	sc584|g24349
316B/A	TGTGATCAATTGTGAAGACGAA/ATAATCTGCGTGCTCCCTCT	52.4/55.9	227	10	7	0.0129	sc1937|gn
360B/A	CCCAGAAGWCAAACTGATGTATGC/CCAGTGTTAGGTTTAAGCGTGCAG	55.9/58.2	287	8	14	0.0068	sc741|g29697
440B/A	ATCGTTCGTGGTCATTGGTTTGCC/ATGTGCGATGGCACCATGGAAATG	60.2/60.5	231	27	7	0.0411	sc299|g8719
449B/A	GATTCGTCGTCGTGTCAATG/CCACGGCAAACTTAGCAAAT	53.8/54.0	248	2	3	0.0018	sc719|g33222
469B/A	CTGATAGACCGCTATGGAACGTAG/AGGCTGAACTGCGAGAAAGTGGT	56.4/60.9	441	15	9	0.0078	sc672|g27554
503B/A	CATCGCCAAGCAACACTTCTCCAT/AGGTTGGAAAGGAGTACGAGCTGA	59.9/59.6	313	10	17	0.0062	sc983|g36290
524B/A	GCAAGCCATACATGTGCCAGATTTGC/GCATTGATAGTGTTCTGATGCTGCCG	61.0/60.1	277	4	7	0.0033	sc689|g27494
550B/A	TCCATGTTTCTACGCAGTGAGG/TGCTCTGCAAGTGATGTTCATTGT	56.9/57.4	330	7	5	0.0062	sc1204|g40050
586B/A	CACTACCTTCTTCGAGGTGTGCAA/TCACAGCAGGATCATCACCGAACA	58.8/60.3	222	1	2	0.0183	sc67|g3272
590B/A	GTCAAGTGTATACGATTTCAACAAG/GGAAGGCACCAGTGACTACAAT	52.3/57.0	231	8	9	0.0095	sc977|g11464
632B/A	TGGGATAAATCGAAATCTGAGAGGA/GGTGCGTTTCACAGATTTAGCAGTCC	55.4/60.0	256	6	5	0.0151	sc411|g17731
676B/A	CCCTGGTTTACTCTCTCTGGTCAA/CCTTCGGCCGTGTTACGTTGTTT	58.0/60.2	257	15	10	0.0104	sc1159|gn
677B/A	CTGGKATGCTRAATTGTGTTCTGC/GGCCACCTCTTCAAATTCTGCGAT	56.6/59.8	184	5	10	0.0062	sc1616|gn
712B/A	GTTGAAATATCTAAACATTGCTGCTGA/CGTGGCTCAATTTAATGGTGACGG	54.3/58.5	249	2	3	0.0024	sc436|g18214
Total			6886	203	48	0.0079	

1 The primer set was labeled for the contig, followed by B and A for right and left primers for the contig. More primer information is available in Table S2 of Fu and Peterson (2012).

2 Tm, annealing temperature; CL, contig length flanking by the primer set; Ts, the total number of segregating sites; Nh, the number of haplotypes observed; π, the estimate of nucleotide diversity; Scaffold|GO, the scaffold number and gene annotation number available at http://WWW.linum.ca, and gn means no gene annotation found for the contig.

### Sequence analysis

All sequencing products were assembled with Vector NTI Suite's ContigExpress v9.0.0 (Invitrogen, Carlsbad, CA) and aligned using MUSCLE v3.6 (Edgar 2004). Aligned sequences with required length and quality were deposited into GenBank under accessions JN845641--JN846695 and JN861766 and those without are given in Table S1. Population genetic analyses of aligned DNA sequences were performed using DnaSP program (Librado and Rozas 2009). Several measures of sequence variation were obtained, and they are the number of segregating sites, haplotype number, nucleotide diversity (π; Tajima 1983), the signal of selection (i.e., deviation from neutrality; Tajima 1989; Fu and Li 1993), and the frequency of recombination (i.e., the minimum number of recombination events; Hudson and Kaplan 1985). The comparative diversity analyses were also done for different loci and various *Linum* groups. Haplotype analyses with and without gaps were performed using the DnaSP program. The positions of SNPs and indels for each haplotype were generated.

The genetic relationships of the 48 *Linum* samples were analyzed based on concatenated sequences using the Bayesian Markov chain Monte Carlo approach available in the BEAST v1.4 (Drummond and Rambaut 2007), as the concatenation approach tends to yield more accurate trees than the consensus one (Gadagkar et al. 2005). The maximum clade credibility (MCC) phylogenies were generated with a relaxed uncorrelated lognormal clock and with tree priors as constant size, expansion, or exponential growth. The substitution model was under an HYK model with gamma distribution for site heterogeneity. The rest of the options were applied with default values. This Bayesian approach should yield more informative phylogeny, as it directly calculates ultrametric phylogenies based only on observed data and model parameters and incorporates both the branch-length errors and the topological uncertainties (Rutschmann 2006). For comparison, the distance-based NeighborNet (Bryant and Moulton 2004) of the 48 samples was also generated using the SplitsTree4 (Huson and Bryant 2006) with the default options of Uncorrected_P and EqualAngle. The NeighborNet should display detailed reticulations where recombination may occur and yield more information for understanding the genetic relationships.

The optimal genetic structure of the 48 samples was also inferred based on concatenated sequences with a model-based Bayesian method available in the program STRUCTURE v2.2.3 (Pritchard et al. 2000; Falush et al. 2007). The STRUCTURE program was run 20 times for each subpopulation (*K*) value, ranging from 2 to 10, using the admixture model with 10,000 replicates for burn-in and 10,000 replicates during analysis. The final population subgroups were determined based on (1) likelihood plot of these models, (2) the change in the second derivative (Δ*K*) of the relationship between K and the log-likelihood (Evanno et al. 2005), and (3) stability of grouping patterns across 20 runs. For a given *K* with 20 runs, the run with the highest likelihood value was selected to assign the posterior membership coefficients to each accession. A graphical bar plot was then generated with the posterior membership coefficients. To assess the consistency of structural inference, an additional analysis was also made with the Bayesian method available in the BAPS software (Corander et al. 2008). Individual samples were clustered using the model for linked (or concatenated) markers and 20 replicate runs of the algorithm with the upper-bound values (*K*) for the number of clusters ranging between 2 and 10.

An analysis of molecular variance (AMOVA) was performed based on concatenated sequences using Arlequin v3.01 (Excoffier et al. 2005) to quantify nucleotide variation between species and among various groups and inferred clusters of *Linum* accessions. Three models of genetic grouping were considered: pale flax versus cultivated flax; five groups of pale flax and cultivated flax; and four clusters of Linum samples inferred using the BEAST program. The significance of variance components and intergroup genetic distances (or pairwise group *F*st) for each model was tested with 10,000 random permutations. The analysis also generated group-specific *F*st values in each model.

### Coalescent simulation for bottleneck

The intensity of the bottlenecks associated with flax domestication was estimated following the procedures described in Haudry et al. (2007) using Hudson's ms program (Hudson 2002). The procedures applied simple demographic model of reduction in effective population size, assumed that an ancestral population experienced an instantaneous change in effective population size many generations ago (*t*) and no population expansion after the bottleneck. The bottleneck intensity α was defined as the ratio of the wild population size (*N*_a_) to cultivated population size (*N*_p_). Higher values of α correspond to more severe bottlenecks. The model had five parameters (*N*_a_, *N*_p_, τ, θ_wild_, and 4*Nc*) and the last three are the time after the bottleneck, the ancestral nucleotide diversity, and the population recombination at the locus, respectively. In this simulation, we assumed *N*_a_= 30,000 similar to those predicted in wheat and barley domestication (Badr et al. 2000; Haudry et al. 2007) and cultivated flax had gone through (*t =*) 9000 generations (or years) of domestication, so that τ= 0.15α. The estimates of θ_wild_ and 4*Nc* at each locus for pale flax were obtained in this study. A set of 19 values of α was explored on a grid ranging from 1 (no reduction in effective population size) to 10 (severe reduction in effective population size), with 5000 simulations and an effective sequence length of 184 to 441 bp. The proportion of 5000 runs that simulated π is within 20% of the observed π was calculated for each α value for each of five domestication groups (dehiscent, fiber, oil, winter, and all cultivated flax samples). The average bottleneck intensity for each domestication group was estimated following Haudry et al. (2007) by calculating a multilocus likelihood as the product over 24 locus-specific likelihoods and maximizing the multilocus likelihood with respect to α. A 95% confidence interval was also constructed around the estimate of α by determining the value of α at which the log-likelihood value was 2 log-likelihood units lower than the maximized likelihood.

## Results

### Nucleotide polymorphism

The Sanger resequencing generated a total of 1152 sequences of 24 DNA fragments for 48 *Linum* samples (Table 2). These DNA fragments represented 24 unlinked loci sampled across the flax genome. Sixteen fragments were associated with predicted gene functions, mainly with different proteins, but were not fully annotated for further diversity analysis. The DNA fragments varied in length ranging from 184 to 441 bp and averaging 289 bp. The total length of 24 concatenated sequences for each sample was 6886 bp. The number of segregating sites per DNA fragment ranged from 1 to 27 and averaged 8.5. The number of haplotypes detected per DNA fragment ranged from 2 to 17 and averaged 2.

For pale flax, the number of segregating sites per fragment ranged from 1 to 25 and averaged 6.4 and the estimated nucleotide diversity ranged from 0.0027 to 0.0603 and averaged 0.0108 (Table 3). For all the cultivated flax samples, the number of segregating sites per fragment ranged from 1 to 25 and averaged 7.0 and the estimated nucleotide diversity ranged from 0.0008 to 0.0351 and averaged 0.0076. For all 24 loci, the overall nucleotide diversity was larger for pale flax (0.0097) than for cultivated flax (0.0071). For the four groups of cultivated flax, large variation in nucleotide polymorphism was observed (Table 4). The number of segregating sites per fragment ranged from 0 to 25 and averaged 4.6 for the dehiscent flax; 0 to 7 and 2.5 for the fiber flax; 0 to 22 and 4 for the oil flax; and 0 to 22 and 4.5 for the winter flax. The estimated nucleotide diversity ranged from 0 to 0.0522 and averaged 0.0075 for the dehiscent flax; 0 to 0.0157 and 0.0033 for the fiber flax; 0 to 0.0217 and 0.0051 for the oil flax; and 0 to 0.0578 and 0.0072 for the winter flax. For all 24 loci, the highest estimated nucleotide diversity was 0.0071 for the dehiscent flax, followed by the winter flax (0.0069), the oil flax (0.0053), and the fiber flax (0.0034).

**Table 3:** Comparative nucleotide polymorphisms between pale flax and cultivated flax at 24 sampled genomic regions.

Primer	*S*^1^	π^1^	*D*^1^	*D*/*F*^1^	*R*m^1^	*S*	π	*D*	*D*/*F*	*R*m
					*Pale flax*					*Cultivated flax*
031B/A	9	0.0102	0.454		0	4	0.0040	1.126		0
049B/A	3	0.0042	1.152		0	14	0.0089	–0.343		1
071B/A	10	0.0104	–0.754		4	11	0.0070	–0.739		4
145B/A	2	0.0030	1.642		0	4	0.0049	1.967#	ns|#	1
151B/A	9	0.0167	1.310	^*^|#	0	3	0.0042	0.959		0
204B/A	1	0.0027	1.303		0	6	0.0056	–0.344		0
221B/A	11	0.0099	–0.281		1	10	0.0105	1.458		0
242B/A	5	0.0072	0.931	#|ns	2	4	0.0056	0.992		2
246B/A	4	0.0041	–0.943		0	3	0.0026	–0.158		0
281B/A	4	0.0053	0.264		0	7	0.0063	0.200		0
316B/A	9	0.0251	1.219		0	6	0.0078	0.627		0
360B/A	8	0.0121	0.026		1	7	0.0055	–1.013		3
440B/A	25	0.0603	1.585		0	25	0.0353	0.503		0
449B/A	2	0.0043	1.642		0	2	0.0008	–1.102		0
469B/A	6	0.0065	1.455		0	15	0.0088	–0.202		0
503B/A	4	0.0048	0.204		1	9	0.0063	–0.510		3
524B/A	2	0.0027	0.222		0	4	0.0034	–0.041		2
550B/A	7	0.0065	–0.584		0	5	0.0051	1.057		0
586B/A	2	0.0136	–0.184		0	1	0.0125	0.976		0
590B/A	5	0.0116	0.981	#|ns	0	7	0.0068	–0.158		1
632B/A	7	0.0096	–0.318		0	6	0.0161	2.778^*^^*^	ns|^*^^*^	0
676B/A	13	0.0162	–0.458		1	8	0.0081	–0.286		0
677B/A	4	0.0087	0.143		1	5	0.0054	–0.605		2
712B/A	2	0.0032	0.120		0	1	0.0023	1.643		0
Total	154	0.0097	0.555		11	167	0.0071	0.323		19

1 Four polymorphism parameters are *S* for the number of segregating sites; π, the nucleotide diversity (Tajima 1983); *D*, selection test by Tajima's *D* (Tajima 1989); *D*/*F*, significant results obtained by Fu and Li's D^*^ and Fu and Li's F^*^ (Fu and Li 1993); *R*m, the minimum number of recombination events (Hudson and Kaplan 1985); and significance of test, ns *P* > 0.05, # *P*≈ 0.05,^*^P < 0.05,^*^^*^P < 0.01.

**Table 4:** Comparative nucleotide polymorphisms among four groups of cultivated flax at 24 sampled genomic regions.

Primer	*S*^1^	π^1^	*D*^1^	*D*/*F*^1^	*R*m^1^	*S*	π	*D*	*D*/*F*	*R*m
				*Dehiscent group*					*Fiber group*	
031B/A	0	0.0000	nd		nd	0	0.0000	nd		nd
049B/A	9	0.0116	0.545	^*^|ns	0	1	0.0006	–1.112		0
071B/A	10	0.0140	0.138		0	2	0.0027	0.222		0
145B/A	2	0.0019	–0.448		0	4	0.0057	1.7724#	ns|#	0
151B/A	3	0.0053	0.458		0	0	0.0000	nd		nd
204B/A	3	0.0033	–1.448		0	3	0.0042	–0.431		0
221B/A	7	0.0101	1.411		0	7	0.0089	0.900		0
242B/A	2	0.0026	0.069		0	6	0.0064	–0.366		1
246B/A	2	0.0026	–0.448		0	1	0.0021	1.303		0
281B/A	3	0.0056	1.601		0	3	0.0037	0.021		0
316B/A	0	0.0000	nd		nd	0	0.0000	nd		nd
360B/A	1	0.0011	–1.055		0	4	0.0033	–1.245		0
440B/A	25	0.0522	0.248		0	0	0.0000	nd		nd
449B/A	1	0.0017	0.334		0	0	0.0000	nd		nd
469B/A	8	0.0074	0.258		0	6	0.0040	–1.103		0
503B/A	9	0.0122	–0.060		1	5	0.0046	–0.783		1
524B/A	3	0.0027	–1.448		0	2	0.0039	1.743#		1
550B/A	4	0.0065	1.697	ns|#	0	0	0.0000	nd		nd
586B/A	4	0.0108	0.182		1	1	0.0029	0.820		0
590B/A	3	0.0040	–0.813		0	0	0.0000	nd		nd
632B/A	1	0.0025	1.444		0	7	0.0157	2.383^*^^*^	^*^|^*^^*^	0
676B/A	6	0.0110	1.022		0	5	0.0063	–0.329		0
677B/A	4	0.0094	0.081		1	2	0.0024	–1.401		0
712B/A	1	0.0025	1.444		0	1	0.0021	1.303		0
Total	111	0.0071	0.345		3	60	0.0034	0.276		3
					*Oil group*				*Winter group*	
031B/A	0	0.0000	nd		nd	1	0.0006	–1.112		0
049B/A	6	0.0081	1.108		0	5	0.0071	1.334	#|ns	1
071B/A	3	0.0032	–0.507		0	3	0.0039	0.097		0
145B/A	4	0.0059	1.953#	ns|#	0	4	0.0055	1.591	ns|#	0
151B/A	1	0.0008	–1.112		0	1	0.0019	0.820		0
204B/A	1	0.0021	0.820		0	4	0.0043	–1.245		0
221B/A	7	0.0068	0.025	^*^|ns	0	7	0.0089	1.356	^*^|#	0
242B/A	3	0.0042	–0.130		0	6	0.0073	0.198		2
246B/A	1	0.0021	1.303		0	1	0.0018	0.820		0
281B/A	7	0.0094	0.329		0	4	0.0052	0.143		0
316B/A	1	0.0009	–1.112		0	0	0.0000	nd		nd
360B/A	9	0.0115	0.026		3	7	0.0091	–0.348		0
440B/A	22	0.0217	–2.053^*^^*^	^*^^*^|^*^^*^	0	22	0.0578	2.405^*^^*^	^*^^*^|^*^^*^	0
449B/A	0	0.0000	nd		nd	2	0.0016	–1.401		0
469B/A	8	0.0100	2.093^*^	^*^|^*^^*^	0	11	0.0089	–0.255		0
503B/A	2	0.0026	0.526		1	4	0.0053	0.686		1
524B/A	2	0.0020	–0.691		0	4	0.0044	–0.521		0
550B/A	5	0.0054	0.024	#|ns	0	5	0.0054	0.024	#|ns	0
586B/A	0	0.0000	nd		nd	0	0.0000	nd		nd
590B/A	0	0.0000	nd		nd	4	0.0055	–0.400		0
632B/A	7	0.0154	2.041^*^	^*^|^*^^*^	0	6	0.0175	2.1482^*^	#|^*^^*^	0
676B/A	4	0.0038	–1.667#	#|#	0	4	0.0037	–1.245		0
677B/A	2	0.0040	–0.184		0	2	0.0042	0.019		0
712B/A	1	0.0022	1.464		0	1	0.0021	1.303		0
Total	96	0.0053	0.003		4	108	0.0069	0.736		4

1 Four polymorphism parameters are *S* for the number of segregating sites; π, the nucleotide diversity (Tajima 1983); *D*, selection test by Tajima's *D* (Tajima 1989); *D*/*F*, significant results obtained by Fu and Li's D^*^ and Fu and Li's F^*^ (Fu and Li 1993); *R*m, the minimum number of recombination events (Hudson and Kaplan 1985); and significance of test, ns *P* > 0.05, # *P*≈ 0.05,^*^P < 0.05,^*^^*^P < 0.01; nd, no data.

### Selection, recombination, and bottleneck

For pale flax, no significant deviation from neutrality measured with Tajima's *D* was detected for any loci assayed, but two deviations from neutrality (one significant and one marginally significant) were observed for cultivated flax (Table 3). However, if based on Fu and Li's D* and F* tests, there were three possible significant deviations from neutrality for pale flax. For the four groups of cultivated flax, the largest number of significant (and/marginally significant) tests for deviation from neutrality based on Tajima's *D* was 5 for the oil flax, followed by the winter flax (2), the fiber flax (2) and the dehiscent flax (0) (Table 4). If based on Fu and Li's D* and F* tests, the largest number of significant (and/marginally significant) tests for deviation from neutrality was 7 for the oil flax, followed by the winter flax (6), the fiber flax (2) and the dehiscent flax (2).

The recombination analysis performed with the DnaSP program revealed large variation in recombination frequency with respect to species and group (Tables 3 and 4). The total number of recombination events at the 24 loci was 11 for the 10 pale flax samples and 19 for the 38 cultivated flax samples. The total number of recombination events at the 24 loci was four for the oil and winter flax and three for the dehiscent and fiber flax.

The coalescent simulations assuming a simple demographic model with observed values of related parameters revealed the extent of domestication bottleneck ranging from 1.5 to 2 for the four groups of cultivated flax and the whole cultivated flax samples (Table 5). Specifically, based on the estimated π for each group, the bottleneck intensity was estimated to be 2 for the oil flax group and 1.5 for the other groups. The estimates of the 95% confidence interval were also large, ranging from 1 to 3.0, depending on the group of interest.

**Table 5:** The estimates of bottleneck intensity by coalescent simulations and proportional nucleotide variations among pale flax and four groups of cultivated flax obtained from the analysis of molecular variance at 24 sampled genomic regions.

			Pairwise group *F*st^2^			
Group (size)	Intensity of bottleneck^1^	Group-specific *F*st	Dehiscent	Fiber	Oil	Winter
Pale (10)		0.222	0.254^*^^*^^*^	0.276^*^^*^^*^	0.216^*^^*^^*^	0.162^*^^*^
Dehiscent (8)	1.5 (1.2–1.7)	0.254		0.500^*^^*^^*^	0.424^*^^*^^*^	0.351^*^^*^^*^
Fiber (10)	1.5 (1.2–1.9)	0.299			0.103^*^^*^	0.161^*^^*^
Oil (10)	2.0 (1.1–2.4)	0.276				0.056ns
Winter (10)	1.5 (1.0–3.0)	0.254				
Cultivated (38)	1.5 (1.0–1.9)					
Mean /range		0.261/0.222–0.299				

1 The values in parentheses represent the 95% confidence intervals, estimated with 2 log-likelihood units lower than the maximum likelihood estimate of bottleneck intensity.

2 The significance of test with ns *P* > 0.05,^*^^*^P < 0.001,^*^^*^^*^P < 0.0001.

### Genetic relationship

**Figure 1:**
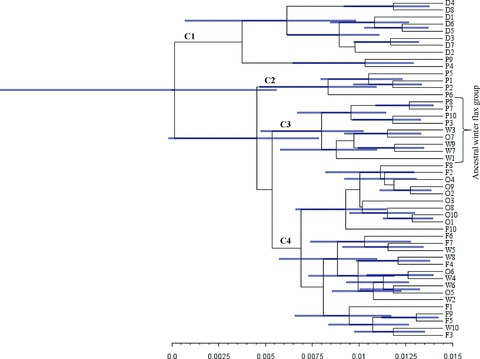
The maximum clade credibility trees of the 48 *Linum* accessions representing pale flax and four groups of cultivated flax obtained by the BEAST program based on 24 sampled genomic regions. The node bar for Length_95%_HPD is shown. The first capital letter of the sample label represents the flax group (Table 1). Four major clusters (C1–C4) are labeled on the branches. The ancestral winter flax group is highlighted.

The BEAST program generated three MCC trees for the 48 *Linum* samples with three tree priors as constant size, expansion, and exponential, respectively. The phylogenies with the first two tree priors were exactly the same, although estimated branch lengths (or evolutionary rates) varied. The MCC tree with tree prior as expansion mirrored more closely with the NeighborNet by SplitsTrees4 described below. The MCC tree with tree prior as exponential had a cluster with mixed memberships from pale, winter, oil, dehiscent flax samples and was slightly less compatible with the NeighborNet. Figure 1 showed the MCC tree of the 48 *Linum* samples obtained with the tree prior as expansion. The cluster at the top (C1) consisted of eight dehiscent flax samples and two pale flax samples (P9 and P4), followed by a small cluster (C2) of four pale flax samples (P5, P1, P2, P6). The next cluster down (C3) had four pale flax samples (P8, P7, P10, P3), four winter flax samples (W3, W9, W7, W1), and one oil flax sample (O7). The bottom large cluster (C4) consisted of 25 samples representing fiber, oil, and winter flax. The detailed members of each cluster are given in Table 1.

Clearly, the winter flax samples were divided into two groups; one with C4 mixed with other cultivated flax samples and the other with C3 closer to some pale flax. The cluster C3 is unique and thus named as the ancestral winter flax group (Fig. 1), as this winter flax group displayed substantial ancestral polymorphism with and close relatedness to pale flax samples. The four ancestral winter flax samples were originated from Afghanistan, Syria, Turkey, and Egypt; the oil flax sample came from Iran; and four pale flax samples were collected from Samsun, Kastamonu, Zonguldak, and Trabzon regions of Turkey. All the members of the ancestral winter group were associated with three *sad2* haplotypes (IX, X, XI; Table 1). Quantifying nucleotide variation among four inferred clusters of *Linum* samples revealed a significant (*P* < 0.0001) differentiation among these inferred clusters, which explained 35.8% nucleotide variation. The ancestral winter flax group (C3) was significantly differentiated from the other three clusters (Table 6).

**Table 6:** The AMOVA results at 24 sampled genomic regions for four clusters of *Linum* accessions inferred by the BEAST program.

		Pairwise cluster *F*st^2^		
Cluster (size)^1^	Cluster-specific *F*st	C2	C3	C4
C1 (10)	0.337	0.306^*^^*^	0.317^*^^*^	0.392^*^^*^
C2 (4)	0.373		0.340^*^	0.369^*^^*^
C3 (9)	0.339			0.366^*^^*^
C4 (25)	0.371			
Mean/range	0.355/0.337–0.373			

1 The members of each cluster are given in Table 1. C3 represents the ancestral winter flax group.

2 The significance of test with^*^P < 0.001,^*^^*^P < 0.0001.

**Figure 2:**
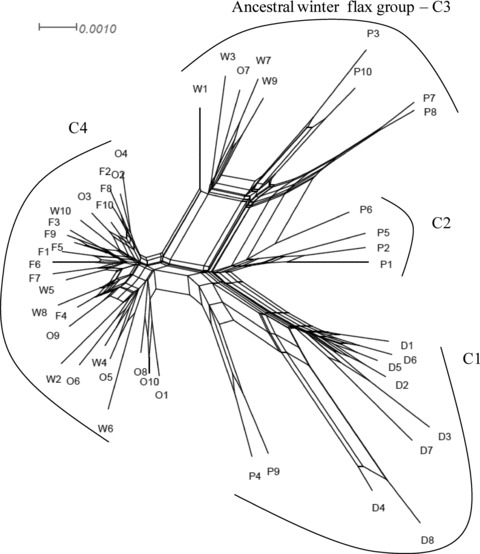
The NeighborNets of the 48 *Linum* accessions representing pale flax and four groups of cultivated flax obtained by the SplitsTree4 program based on 24 sampled genomic regions. The first capital letter of the sample label represents the flax group (Table 1). The four major clusters (C1–C4) obtained by the BEAST program (Fig. 1) are outlined and C3 is the ancestral winter flax group.

The NeighborNet of the 48 *Linum* samples obtained (Fig. 2) revealed essentially the same patterns of genetic relationships as those in the MCC tree, but with higher resolution for recombination at the individual sample level. The winter flax samples also were divided into two clusters; one with six members was closely related to the oil and fiber flax samples, and the other with four members was closely related to the oil flax sample from Iran and became closer to four pale flax samples. The whole dehiscent group was closely related to the pale flax samples. The fiber flax samples were placed in a cluster with a large articulation and mixed with the oil flax samples.

### Genetic structure

**Figure 3:**
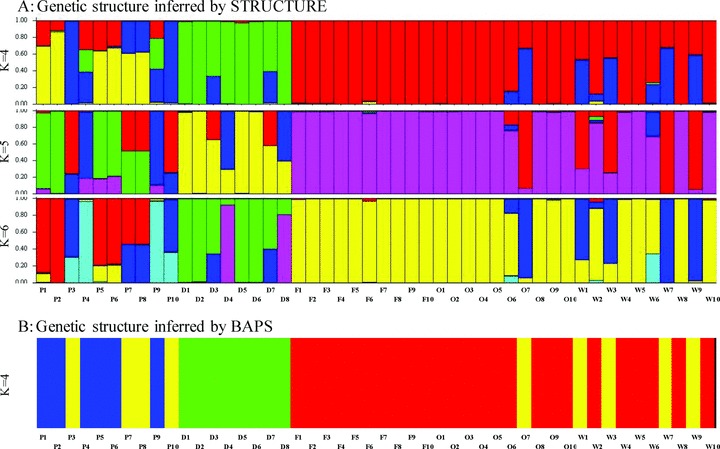
Genetic structure and ancestry of the 48 *Linum* accessions representing pale flax and four groups of cultivated flax inferred by STRUCTURE (**A**) and BAPS (**B**) based on 24 sampled genomic regions. Five optimal clusters were inferred by STRUCTURE and four optimal clusters by BAPS. Each sample is labeled on the bottom of graphical bars and the first capital letter of the sample label represents the *Linum* group (see Table 1). Note that the corresponding clusters may have different colors.

The model-based inference of genetic structure within the 48 *Linum* accessions by STRUCTURE considered *K*= 2--10 clusters and revealed five optimal clusters with the highest log-likelihood value of –3400.3. The inference of the optimal number of clusters gained further support from the change in the second derivative (Δ*K*) of the relationship between *K* and the log-likelihood (results not shown). Figure 3A shows the inferred genetic structure and ancestry for the 48 *Linum* samples for three runs with the highest log-likelihood values under *K*= 4, 5, and 6. Clearly, the changes of ancestry between *K*= 4 and 5 and between *K*= 5 and 6 were not extensive. Under *K*= 5 (i.e., the five optimal clusters), the 10 pale flax samples were divided into four ancestral groups; one was species specific and three were shared with the dehiscent, winter, or oil flax samples. Interestingly, the largest number of *Linum* samples (6) sharing ancestry with the pale flax was observed in the winter flax, followed by those in the dehiscent flax (4), the oil flax (2), and the fiber flax (1).

The model-based inference of genetic structure by BAPS revealed only four optimal clusters with little mixed ancestry (Fig. 2B). The pale flax was divided into two clusters, one of which was shared with one oil and four winter flax samples. The dehiscent flax formed one unique cluster, while the other cultivated flax samples formed another cluster. Clearly, a large number of fiber, winter, and oil flax samples were genetically related, except for those in the cluster mixed with pale flax.

Characterization of a priori genetic structure present in the 48 *Linum* samples using the Arlequin program revealed 15.7% nucleotide variation present between pale flax and cultivated flax and 26.1% residing among five *Linum* groups (one pale flax and four cultivated flax groups). The pale flax samples appeared to have the smallest group-specific *F*st value (0.222), followed by the dehiscent and winter group (0.254), the oil group (0.276), and the fiber group (0.299) (Table 5). The pairwise group differentiations were large, ranging from 0.056 (for the group pair oil flax and fiber flax) to 0.500 (for the group pair dehiscent flax and fiber flax) and averaging 0.250 (Table 5).

## Discussion

This study represents the first large resequencing effort to sample *Linum* genomic regions for the assessment of flax nucleotide diversity and inference of flax domestication history. The effort generated an interesting finding of an ancestral winter group of cultivated flax that displayed close relatedness to pale flax. A related diversity analysis revealed an overall 27% reduction of nucleotide diversity in cultivated flax when compared with the pale flax. Additional analyses showed that recombination frequently occurred at these sampled genomic regions, but the signal of selection and bottleneck was relatively weak. These findings provide some insight into the impact and processes of flax domestication and are significant for expanding our knowledge about early flax domestication, particularly for winter hardiness.

Few estimates of nucleotide diversity are available in *Linum* species (Fu et al. 2012). This study generated a new, useful set of nucleotide diversity estimates for two *Linum* species. The estimates at the 24 sampled genomic regions were higher (0.0071--0.0097) than those at the *sad2* locus (0.0017--0.0052). For cultivated flax, the trait-specific group with the highest estimate of nucleotide diversity was dehiscent, followed by winter, oil, and fiber flax (Table 4). For the *sad2* locus, however, the trait-specific group with the highest estimate of nucleotide diversity was winter, followed by oil, fiber, and dehiscent flax (Fu et al. 2012). Overall, the estimates of nucleotide diversity for these two species appeared to be compatible with those reported for outcrossing crops such as maize (Wright et al. 2005) and much higher than those for other inbreeding species such as wheat and barley (e.g., see Table 3 of Haudry et al. 2007). These findings are surprising, as a self-fertilization rate of 95% or higher was reported in cultivated flax (Robinson 1937). However, the mating system and gene flow in the wild populations of pale flax remain unknown, although two distinct genetic backgrounds were detected in pale flax accessions collected from Turkey and associated with site elevation and longitude (Uysal et al. 2010, 2012). Also, it is possible that the high estimates of nucleotide diversity reflect the effect of sampling genomic regions only with the most polymorphism.

The impact of domestication on cultivated flax seems to be only moderate at the sampled genomic regions. First, the overall reduction of nucleotide diversity (27%) in cultivated flax with respect to pale flax was not large, when compared with those for the inbreeding species such as wheat and barley (e.g., see Table 3 of Haudry et al. 2007, but also see Kilian et al. 2007). When trait-specific groups of cultivated flax are considered, the impact appears to be large, ranging from 27% to 65% and is compatible with those previously reported (Haudry et al. 2007). Second, the overall selection at these genomic regions was relatively weak, as significant deviations from neutrality were not extensive across all the genomic regions assayed (Tables 3 and 4). Third, the estimated intensities of domestication bottleneck for cultivated flax and trait-specific groups were also weak, ranging from 1.5 to 2, implying that the effective population size after domestication was onefold smaller than the effective population size in the wild progenitor population. These levels of bottleneck were considerably weak, when compared with those inferred in wheat (3; Haudry et al. 2007) and rice (3.5; Li et al. 2011). However, more extensive coalescent simulations for bottleneck are desirable with an expanded genomic coverage and outgroup sequence.

The BEAST program clustered the assayed winter flax samples into two groups, one of which was closely related to pale flax (Fig. 1). This ancestral winter flax group was significantly differentiated from the other three clusters including the other group of winter flax (Table 6). The genetic division in the winter flax samples gained further support not only from the NeighborNet analysis with the SplitsTree4 (Fig. 2), but also from the Bayesian inferences of genetic structure with the STRUCTRUE and BAPS programs (Fig. 3). The ancestral winter flax group was consistently formed with compatible inferences of ancestry for each member, although these Bayesian inferences varied between two methods such as in the optimal cluster number. Also, the STRUCTURE program seems to yield more information on ancestry for the ancestral winter flax group than the BAPS program. This may reflect the weakness of the BAPS Bayesian method or the effect due to the violation of linked marker with concatenated unlinked sequences.

The discovery of the ancestral winter flax group provides the first set of genetic evidence for early domestication for flax winter hardiness. As mentioned earlier, winter hardiness and capsular dehiscence are two major characteristics of pale flax (Diederichsen and Hammer 1995; Uysal et al. 2012). Previous genetic studies (Uysal et al. 2010; Fu 2011; Fu et al. 2012) showed that the dehiscent flax displayed more genetic similarity to its pale flax, but the winter flax displayed more genetic similarity to oil and fiber flax. The analysis here revealed the genetic division of the assayed winter flax samples; one group displayed more genetic similarity to pale flax. This is consistent with our original reasoning that the winter flax may have experienced differential domestication pressure and some of them still carry substantial ancestral polymorphism from pale flax (Charlesworth 2010). In contrast, the fiber flax samples displayed little ancestral polymorphism from pale flax at these genomic regions (see Fig. 3).

Another interesting result associated with the ancestral winter flax group is its inclusion of the oil flax sample from Iran. This result also has some implications. First, it supports the previous reasoning that flax was domesticated initially for oil, rather than fiber, use (Allaby et al. 2005). Second, it is consistent with the reasoning from the *sad2* locus that multiple independent pathways of domestication of flax occurred after the initial domestication for oil use (Fu et al. 2012). Similarly, as cultivated flax was spread into Europe, winter hardiness was improved along with the selection for oil and fiber traits (Maier and Schlichtherle 2011), so that the nonancestral winter flax samples were well mingled with oil and fiber flax samples (Figs. 1 and 2).

Our study could be further improved for more informative inferences with enlarged sampling in various *Linum* groups and genomic coverage. However, extra efforts are still needed to collect pale flax samples from other regions of its species distribution and to assemble more trait-specific groups of flax germplasm (Diederichsen and Fu 2006; Uysal et al. 2012). The effects of genomic sampling cannot be completely excluded, as the 24 genomic regions were selected mainly based on the polymorphism. Expanding the genomic coverage would help to minimize such sampling effects. Also, 16 of the 24 genomic regions were associated with functional genes (encoding proteins) and should represent the transcribed regions of the flax genome, but it remains unknown that the detected polymorphism was truly ancestral variation from pale flax with respect to winter hardiness. Answering this question would require further investigation of genes or genomic regions knowingly associated with winter hardiness, but such genomic resources currently are still lacking.

The findings presented here are encouraging for searching clues on flax domestication processes. Winter hardiness was among those flax traits that human domesticated early (Fu 2011). This study, along with those companion investigations (e.g., see Uysal et al. 2010; Fu 2011; Fu et al. 2012), helps to establish the early domestication events associated with human selection for oil, fiber, capsular indehiscence, and winter hardiness. These efforts constitute the first important step to unravel the complex sequence and timing of human selection on flax over the last 9000 years. With the development of more informative genomic resources, more ancestral variation will be identified and utilized to establish domestication events. More effort is needed to model, test, and date the domestication paths with these established events. Ultimately, the flax domestication history can be reliably described and better understood.

## Supporting Information

**Table S1.** Aligned Sanger sequences for two contigs (C586and C667) in 48 *Linum* samples.

**Table S2.** List of 24 primer pairs used by Sangerresequencing of 24 contigs representing polymorphic genomic regionsin the 48 *Linum* accessions, along with the polymorphism and gene annotation information.
